# Investigating the Mechanism of Qifenggubiao Granules in COPD Treatment: An Integrated Exploration of Ferroptosis and the Gut–Lung Axis

**DOI:** 10.34133/bmr.0263

**Published:** 2025-10-08

**Authors:** Mianfeng Zheng, Lixin Huang, Haitao Yuan, Zhuoya Li, Yi Wang, Yangjing Su, Zhixin Deng, Ali Chen, Weiguo Zhao, Weiming Wang, Wei Xiao

**Affiliations:** ^1^ Key Laboratory of Glucolipid Metabolic Disorder, Ministry of Education, Guangdong Pharmaceutical University, Guangzhou 510006, China.; ^2^ Department of Nuclear Medicine, First School of Clinical Medicine, The First Affiliated Hospital of Guangdong Pharmaceutical University, Guangzhou 510080, China.; ^3^ Center for Drug Research and Development, Guangdong Provincial Key Laboratory for Research and Evaluation of Pharmaceutical Preparations, Guangdong Pharmaceutical University, Guangzhou 510006, China.; ^4^ Department of Pharmacy, Zhongshan People’s Hospital, Zhongshan 528404, China.; ^5^ Institute of Chinese Materia Medica, Heilongjiang Academy of Chinese Medicine Sciences, Harbin 150036, Heilongjiang, China.

## Abstract

Although an increasing number of studies focus on treating chronic obstructive pulmonary disease (COPD) through the gut–lung axis and immunomodulation, its underlying mechanisms remain poorly understood. Previous research has shown that Qifenggubiao granules (QFGB) exhibit obvious clinical efficacy in treating allergic rhinitis and chronic cough, demonstrating excellent antioxidant and anti-inflammatory properties. However, whether it can alleviate COPD by inhibiting ferroptosis remains unclear. Additionally, its immunomodulatory mechanisms in gut microbiota dysbiosis-related inflammation require further investigation. In this study, we found that QFGB not only suppresses oxidative stress but also inhibits ferroptosis by reducing lipid peroxide levels and increasing the expression of glutathione peroxidase 4 and xCT. The authors also discovered that QFGB significantly alleviates pulmonary dysfunction in COPD animal models by regulating macrophage polarization and remodeling the inflammatory immune microenvironment, thereby suppressing inflammation. Furthermore, 16*S* ribosomal RNA sequencing and quantitative reverse transcription polymerase chain reaction analysis confirmed that QFGB modulates gut microbiota composition and bidirectionally regulates macrophage polarization in lung and intestinal tissues. These findings have been further validated in animal models of inflammatory bowel disease (IBD), demonstrating that QFGB can alleviate inflammation in IBD mice. This study demonstrates that QFGB can not only inhibit oxidative stress and ferroptosis in COPD but also regulate gut microbiota homeostasis and remodel the inflammatory microenvironment by modulating macrophage polarization via the gut–lung axis. The drug alleviates the severity of COPD and promotes functional recovery of the lung–gut organ axis. These findings have been further validated in animal models of IBD, demonstrating that QFGB can alleviate intestinal inflammation in mice.

## Introduction

Chronic obstructive pulmonary disease (COPD), the third leading cause of death globally, imposes a heavy burden on healthcare systems [1,2]. The hallmark features of COPD include chronic airway inflammation and emphysema, resulting in irreversible lung function decline. Concurrently, COPD patients often suffer from comorbid gastrointestinal disorders such as inflammatory bowel disease (IBD) and irritable bowel syndrome, characterized by gut dysbiosis and increased intestinal permeability [3]. Consequently, targeting the gut–lung axis to modulate COPD and its intestinal comorbidities has emerged as a promising therapeutic strategy.

Oxidative stress is present in COPD patients [4]. It is one of the pathogenic mechanisms of COPD. A study showed that the oxidative stress level increased with the progression of COPD [5]. The continued presence of oxidative stress most likely arises from endogenous reactive oxygen species (ROS), including superoxide anions, hydroxyl radicals, and lipid peroxides. The human lung tissue possesses an efficient antioxidant defense system to counteract oxidative stress. However, accumulated ROS lead to excessive depletion of antioxidants such as reduced glutathione (GSH), superoxide dismutase (SOD), and catalase (CAT), resulting in defective antioxidant defense systems in COPD patients [6]. Concurrently, excessive lipid peroxides induce a novel form of iron-dependent programmed cell death—ferroptosis [7]. Glutathione peroxidase 4 (GPX4) is a selenoprotein that plays a crucial role in the GSH–GPX4 pathway, one of the key antioxidant systems. It converts lipid hydroperoxides (LOOH) to lipid alcohols (LOH) to prevent lipid peroxidation. Depletion of GSH inactivates GPX4, leading to massive accumulation of lipid peroxides and ultimately overwhelming ferroptosis. Under the combined effects of oxidative stress and ferroptosis, the lung tissue structure becomes damaged, resulting in emphysema [8,9].

Macrophages play a pivotal role in maintaining host–microbe homeostasis, antigen presentation, immune defense activation, and bacterial infection resistance. Furthermore, they exhibit remarkable plasticity by polarizing into either M1 (pro-inflammatory) or M2 (anti-inflammatory) phenotypes in response to microenvironmental cues [10]. M1 macrophages are associated with inflammatory responses and pathogen defense, while M2 macrophages promote inflammation resolution and tissue repair [11]. Growing evidence indicates that macrophages are significantly increased in the airways, bronchoalveolar lavage fluid (BALF), lung parenchyma, and sputum of COPD patients compared to healthy samples, with a correlation observed between macrophage numbers and the severity of emphysema [12]. The polarization of macrophages into M1 and M2 phenotypes plays a pivotal role in the pathogenesis and disease progression of COPD [13].

The gut and lungs exhibit a bidirectional relationship mediated by interconnected immune and inflammatory regulatory networks. Pulmonary diseases alter the diversity and composition of gut microbiota, leading to dysbiosis [14]. Studies indicate that COPD is linked to gastrointestinal symptoms, including IBD and other disorders. Under the combined effects of pro-inflammatory and oxidative stress mediators, gastrointestinal dysfunction can be induced [15]. Conversely, impaired gut function exacerbates COPD by increasing pro-inflammatory mediators in systemic circulation while compromising nutrient absorption, antioxidant capacity, and pathogen defense [16]. This interplay underscores the importance of extending COPD research beyond the lungs to the gut, offering novel insights into therapeutic strategies targeting the gut–lung axis.

Qifenggubiao granules (QFGB), a proprietary Chinese herbal medicine (National Drug Approval No. B20020410), were developed by the Heilongjiang Academy of Traditional Chinese Medicine. Comprising 6 herbs—*Astragalus membranaceus*, *Acanthopanax senticosus*, *Atractylodes macrocephala*, *Schisandra chinensis*, *Saposhnikovia divaricata*, and *Ophiopogon japonicus*—QFGB is indicated for reinforcing the exterior, strengthening the spleen, tonifying the lungs and kidneys, and alleviating chronic cough during remission. Its formulation and preparation are protected by a national patent (no. ZL 2004 1 0098805.0) and included in the 2020 edition of the Chinese Pharmacopoeia [17]. Our preliminary studies have confirmed the immunomodulatory effects of QFGB and preliminarily elucidated its multi-target, multi-pathway immunoregulatory mechanisms [18].

This study investigates the therapeutic efficacy of QFGB in COPD and its immunomodulatory mechanisms via the gut–lung axis. We first demonstrated the antioxidant and macrophage-modulating effects of QFGB in vitro. Subsequently, we established a COPD mouse model exhibiting emphysema and inflammation to evaluate QFGB’s therapeutic effects on oxidative stress and ferroptosis. Further exploration of the M1and M2 macrophage polarization in the lungs and gut, combined with 16*S* ribosomal RNA (rRNA) sequencing and IBD mouse experiments, elucidated QFGB’s regulatory role in gut microbiota. Our results demonstrate that QFGB can alleviate oxidative stress and ferroptosis in the lungs during COPD, regulate gut microbiota homeostasis, and remodel the inflammatory microenvironment by modulating macrophage polarization via the gut–lung axis, thereby mitigating pulmonary injury and chronic inflammation in COPD.

## Materials and Methods

### Preparation of QFGB-containing serum

The dosage of QFGB was converted from the adult clinical dose to the rat equivalent dose (4.5 g/kg). Five rats were administered the granules via oral gavage once daily for 10 consecutive days. One hour after the final administration, blood was collected from the abdominal aorta and allowed to stand for 30 min. The serum was then separated by centrifugation at 3,000 rpm, filtered through a sterile membrane, and stored at −20 °C for future use [19].

### Drug treatment of H_2_O_2_-stimulated Beas-2B cells

Beas-2B cells were seeded in 96-well plates at a density of 8 × 10^3^ cells per well and incubated for 24 h. The Blank group (control) was treated with Dulbecco’s modified Eagle’s medium (DMEM), the H₂O₂ group was incubated with medium containing 750 μM H₂O₂ for 24 h, while the QFGB (treatment group) group received 750 μM H₂O₂ plus either 2.5% or 5% of QFGB-containing serum in a total volume of 100 μl of medium for 24 h. Subsequently, 10 μl of CCK-8 (Cell Counting Kit-8) reagent (Meilunbio) was added to each well, followed by incubation for 1 h. Finally, the optical density (OD) at 450 nm was measured using a microplate reader (Agilent Technologies Inc.).

### Detection of cellular ROS and iron ions

Beas-2B cells were seeded in 12-well plates at 4 × 10^4^ cells per well and incubated for 24 h. The Blank group received DMEM, the H₂O₂ group was treated with 750 μM H₂O₂ in a total volume of 100 μl of medium for 24 h, and the QFGB group received 750 μM H₂O₂ plus 5% QFGB-containing serum in a total volume of 100 μl of medium for 24 h. After incubation, cells were washed 3 times with phosphate-buffered saline and stained with FerroOrange fluorescent probe (Dojindo, Kyushu, Japan) and 2′,7′-dichlorodihydrofluorescein diacetate (DCFH-DA) probe (Beyotime, Shanghai, China) for 20 min. ROS levels were immediately detected using a Leica microscope (Leica DMi 8, Wetzlar, Germany) and analyzed by ImageJ software.

### Drug treatment of RAW264.7 macrophages with QFGB-containing serum

RAW264.7 macrophages were seeded in 96-well plates at 8 × 10^3^ cells per well and incubated for 24 h. The Blank group received DMEM, while treatment groups received media containing 20%, 10%, 5%, or 2.5% of QFGB drug-containing serum in a total volume of 100 μl for 24 h. Absorbance was measured as described above [20].

### Animal experiments

Male C57BL/6J mice, aged 6 to 8 weeks and weighing 21 to 25 g, were provided by Guangzhou RuiGe Biological Technology Co. Ltd. (China). All animal procedures were approved by the local animal care and use committee of Guangdong Pharmaceutical University (no. gdpulac2022417). A mouse model of emphysema was established by weekly intranasal instillation of 100 μl of a mixture containing 7 μg of lipopolysaccharide (LPS) and 1.2 U of porcine pancreatic elastase (ELT) under isoflurane anesthesia for 4 weeks (*n* = 6). Control animals received 100 μl of saline weekly for 4 weeks. In the treatment group, QFGB solution was administered daily via intragastric gavage, while the control and COPD groups received distilled water. The dexamethasone (DXMS) group received daily intraperitoneal injections of DXMS for 4 weeks.

For the lung function test, mice were anaesthetized with ketamine and xylazine (80 and 10 mg kg^−1^ intraperitoneally, respectively), tracheotomized below the larynx, and intubated with a trachea cannula. Lung function was measured using the Forced Pulmonary Maneuver System (Buxco Research Systems). Following pulmonary function tests, the mice were immediately euthanized and tissues were collected for analysis.

For the IBD model, a total of 18 mice were randomly divided into 3 groups (*n* = 6): control group, model group, and QFGB group. Except for the control group, all mice freely drank 3% dextran sulfate sodium (DSS) solution for 7 d to induce IBD. The QFGB group received daily intragastric administration of QFGB solution at a volume of 0.1 ml/10 g body weight, while both the control and model groups were given distilled water via gavage.

### Histological analysis

Fresh mouse lung and colon tissues were fixed in 4% formaldehyde and sectioned at 5-μm thickness for histological analysis. Lung and colon sections were stained with hematoxylin and eosin (H&E) and evaluated blindly by an experienced pathologist under an optical microscope. Pathological severity was scored as 0, 0.5, 1, 2, or 3, corresponding to none, minimal, mild, moderate, or severe pathology, respectively. To assess lung cell death, terminal deoxynucleotidyl transferase-mediated deoxyuridine triphosphate nick end labeling (TUNEL) staining was performed using a commercial kit (KeyGEN, Nanjing, China).

### Quantitative reverse transcription PCR

Total RNA was isolated with TRIzol reagent (Invitrogen, California, USA) and subsequently reverse-transcribed into cDNA using a commercial kit (Toyobo, Osaka, Japan). Quantitative polymerase chain reaction (PCR) amplification was carried out on a LightCycler 96 system (Roche). Gene expression levels were calculated using the ΔΔ*C*_t_ method, with 18*S* rRNA serving as the internal reference. The sequences of all primers used are provided in Table S1.

### Western blot

Lung tissue homogenates were lysed in radioimmunoprecipitation assay buffer to extract total proteins. The protein samples were resolved on a 10% sodium dodecyl sulfate–polyacrylamide gel and then electrotransferred onto polyvinylidene difluoride membranes. After blocking with 5% nonfat milk in TBST (tris-buffered saline with 0.1% Tween 20) for 1 h at room temperature, the membranes were probed with specific primary antibodies at 4 °C overnight. Following primary antibody incubation, the membranes were treated with horseradish peroxidase-linked anti-rabbit or anti-mouse secondary antibodies (diluted 1:10,000) for 1 h at room temperature. Protein expression levels were quantified relative to glyceraldehyde-3-phosphate dehydrogenase (GAPDH) as the loading control, and band intensities were analyzed using ImageJ software (v1.52).

### Immunofluorescence

Lung specimens were cryosectioned into 5-μm slices and subjected to antigen retrieval with citrate buffer (pH 6.0). Following blocking, the sections were exposed to primary antibodies at 4 °C for 12 to 16 h. Secondary detection was performed using species-matched fluorescent conjugates (Alexa Fluor 594- and Alexa Fluor 488-labeled anti-rabbit immunoglobulin G). Fluorescent signals were visualized and captured using a Zeiss fluorescence imaging system (Zeiss, Oberkochen, Germany). Detailed antibody specifications are provided in Table S2.

### 16*S* rRNA gene sequencing analysis

Fecal DNA was extracted using a DNA extraction kit (Mabio, Guangzhou, China). The V4 region of bacterial 16*S* rRNA was amplified using primers. PCR products were sequenced on an Illumina NovaSeq 6000 platform, and data were processed using QIIME 2 for bioinformatics analysis.

### Quantification and statistical analysis

All data are presented as mean ± SEM and were compared using 2-tailed unpaired Student’s *t* test or one-way analysis of variance (ANOVA) with Holm–Sidak post hoc tests. Significance levels were set at *P < 0.05, **P < 0.01, ***P < 0.001, and ****P < 0.0001. Graphical summaries were created using BioRender.

## Results

### QFGB-containing serum attenuates H_2_O_2_-induced cell damage

**Fig. 1.**
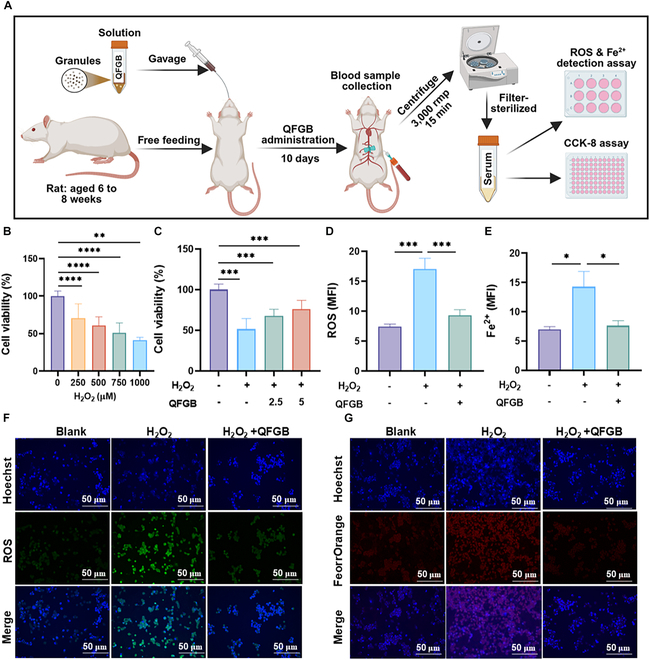
QFGB-containing serum alleviates H_2_O_2_-induced cytotoxicity, oxidative stress, and ferroptosis in Beas-2B cells in vitro. (A) Preparation method of QFGB-containing serum. (B and C) Viability of Beas-2B cells incubated under different conditions for 24 h (*n* = 6). (D and F) ROS levels in Beas-2B cell and mean fluorescence intensity from different groups (*n* = 6). (E and G) Fe^2+^ levels in Beas-2B cell and mean fluorescence intensity from different groups (*n* = 6). Data were evaluated by one-way ANOVA with Holm–Sidak post hoc tests. *P < 0.05, **P < 0.01, and ***P < 0.001. Scale bars, 50 μm.

To investigate QFGB’s effects on oxidative stress and ferroptosis, we prepared drug-containing serum to simulate in vivo conditions (Fig. 1A). CCK-8 assays showed H₂O₂ dose-dependently reduced Beas-2B viability (Fig. 1B), which QFGB-containing serum reversed (Fig. 1C). H_2_O_2_ stimulation induced oxidative stress in cells, and sustained oxidative stress led to excessive production of ROS and iron ion accumulation, ultimately triggering ferroptosis. We quantitatively measured cellular ROS and Fe^2+^ accumulation (Fig. 1D to G), demonstrating that QFGB-containing serum significantly inhibited both H_2_O_2_-induced ROS and Fe^2+^ accumulation. These results showed that the QFGB-containing serum could inhibit oxidative stress and ferroptosis caused by H_2_O_2_ induction in vitro.

### Modulation of LPS-induced M1 polarization in RAW264.7 macrophages by QFGB-containing serum in vitro

**Fig. 2.**
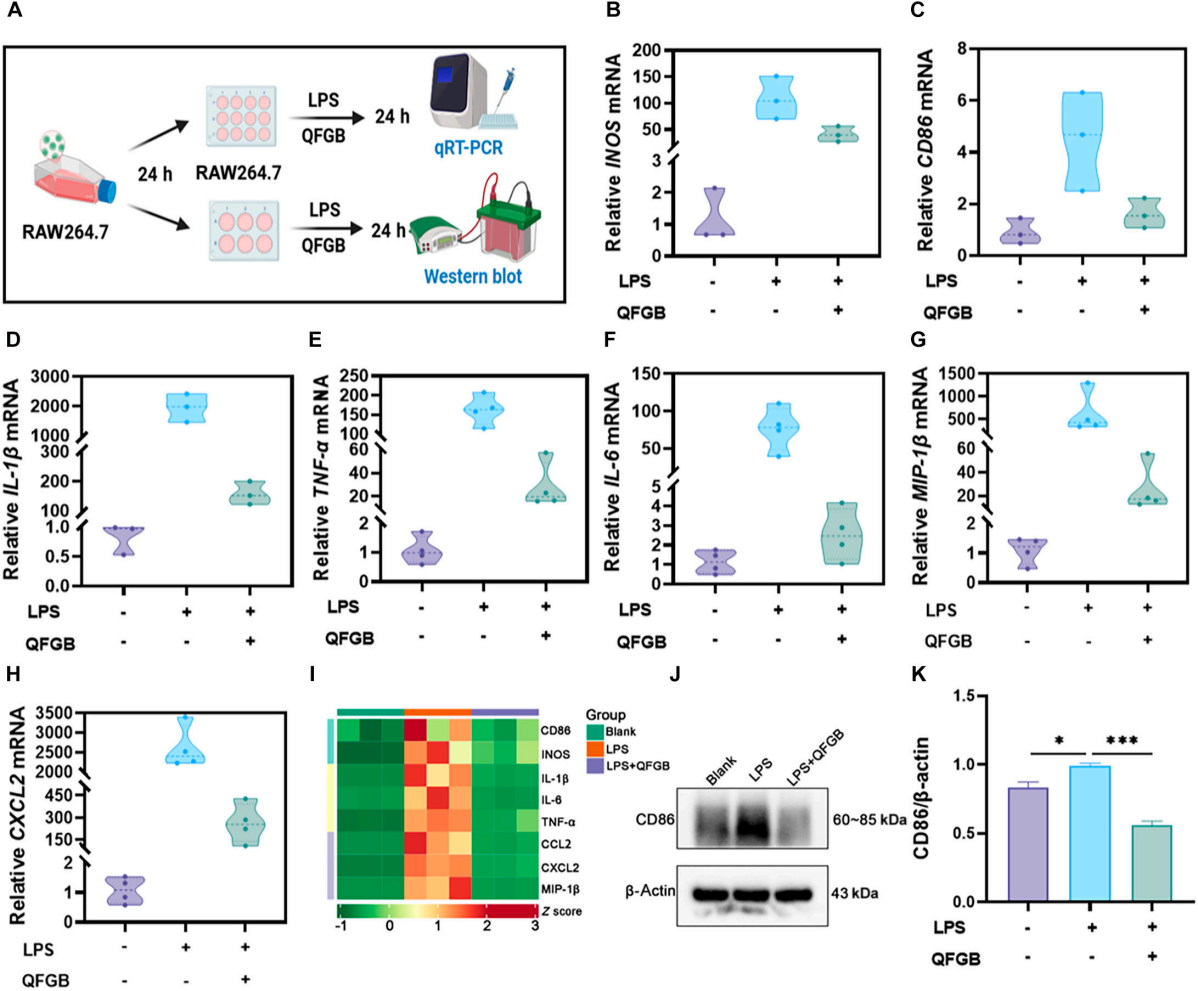
QFGB-containing serum modulates LPS-induced M1 polarization of RAW264.7 macrophages in vitro. (A) Experimental procedures for qRT-PCR and Western blot analysis. (B to H) Relative mRNA expression of M1 macrophage markers and inflammatory cytokines in RAW264.7 cells (*n* = 3 to 4). (I) Clustering heatmap of M1 macrophage markers, inflammatory factors, and chemokines. (J and K) Protein expression levels of CD86 in RAW264.7 cells (*n* = 3). Data were evaluated by one-way ANOVA with Holm–Sidak post hoc tests. *P < 0.05, **P < 0.01, and ***P < 0.001.

QFGB exhibit potent immunomodulatory activities, although their precise mechanisms remain to be elucidated. To investigate their effects on macrophage polarization, we prepared QFGB-containing serum to simulate the in vivo microenvironment and examined its impact on RAW264.7 macrophages (Fig. 2A). We stimulated RAW264.7 macrophages with LPS to induce polarization (Fig. S1A) and concurrently evaluated the effects of QFGB-containing serum on these cells (Fig. S1B). Quantitative reverse transcription PCR (qRT-PCR) analysis of macrophages cotreated with LPS and QFGB-containing serum demonstrated that the serum significantly inhibited LPS-induced M1 polarization, as evidenced by down-regulation of M1 macrophage markers *CD86* and inducible nitric oxide synthase (*INOS*) mRNA and suppression of pro-inflammatory cytokines *TNF-α*, *IL-1β*, and *IL-6* and chemokines *CXCL2*, *MIP-1β*, and *CCL2* (Fig. 2B to I and Fig. S1C). Western blot analysis further confirmed that LPS stimulation significantly increased CD86 protein expression in RAW264.7 macrophages (Fig. 2J and K). These results demonstrate that QFGB-containing serum effectively suppresses LPS-induced M1 polarization of RAW264.7 macrophages in vitro.

### Therapeutic efficacy of QFGB in COPD mice

**Fig. 3.**
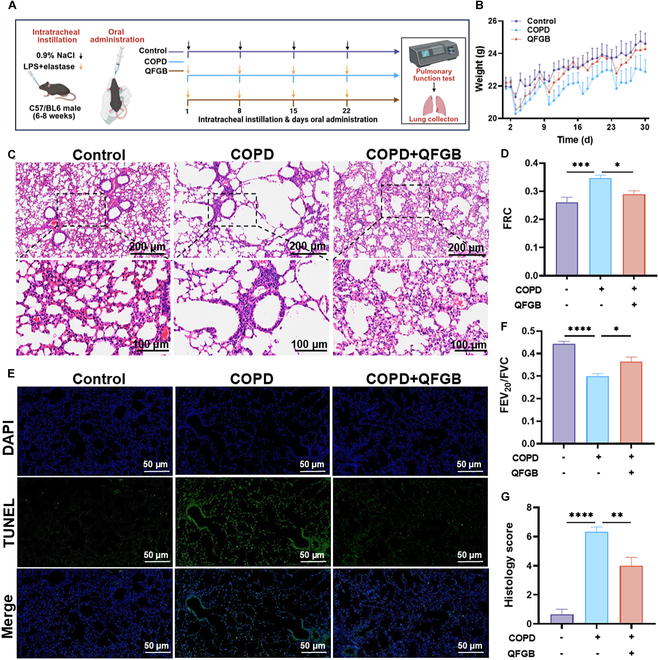
QFGB demonstrates therapeutic efficacy in a murine model of COPD. (A) Schematic diagram of COPD mouse model establishment and drug administration protocol. (B) Line chart of body weight changes in different experimental groups (*n* = 6). (C and G) H&E staining and pathological scoring of lung tissues (*n* = 3). (E) TUNEL staining of lung sections (*n* = 6). (D and F) Lung function test results. FRC, functional residual capacity; FEV_20_/FVC, percentage of the forced expiratory volume in the first 200 ms (*n* = 6). Data were evaluated by one-way ANOVA with Holm–Sidak post hoc tests. *P < 0.05, **P < 0.01, and ***P < 0.001. Scale bars, 200, 100, and 50 μm.

Based on the demonstrated ability of QFGB-containing serum to inhibit oxidative stress and ferroptosis and modulate M1 macrophage polarization, we evaluate the efficacy of QFGB in a COPD emphysema mouse model induced by LPS combined with ELT (Fig. 3A). Body weight analysis demonstrated that QFGB treatment significantly attenuated COPD-induced weight loss (Fig. 3B). Pulmonary function tests demonstrated that QFGB treatment significantly attenuated the COPD-induced decline in lung function (Fig. 3D and F). Histopathological analysis demonstrated that QFGB treatment ameliorated emphysematous changes, including alveolar wall destruction and alveolar fusion, while also attenuating inflammatory cell infiltration (Fig. 3C and G). It is noteworthy that mice treated with DXMS and low-dose QFGB exhibited inferior therapeutic effects (Fig. S2A and B). The TUNEL staining results also showed that QFGB reduced the apoptosis of lung tissue cells in COPD mice after administration (Fig. 3E and Fig. S3). The above results preliminarily demonstrate that QFGB have a therapeutic effect on COPD mice, alleviating lung tissue damage and pulmonary inflammation.

### QFGB attenuates oxidative stress and ferroptosis in COPD mice

**Fig. 4.**
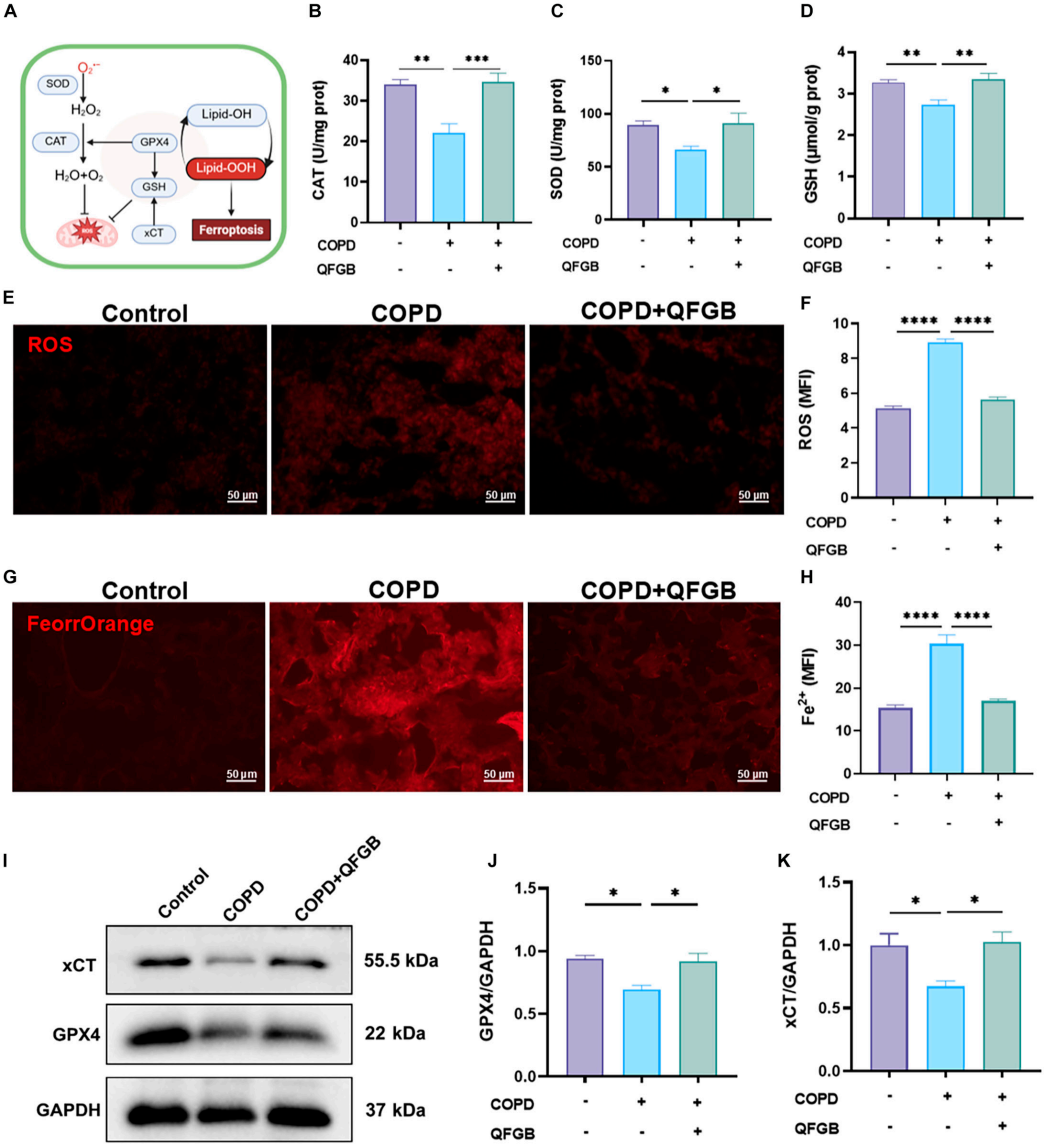
Therapeutic effects of QFGB on pulmonary oxidative stress and ferroptosis in COPD mice. (A) Schematic diagram of oxidative stress and ferroptosis pathways in mice. (B to D) Levels of CAT, SOD, and GSH in mouse lungs (*n* = 6). (E and F) ROS levels in lung tissues of mice and mean fluorescence intensity from different groups (*n* = 6). (G and H) Fe^2+^ levels in lung tissues of mice and mean fluorescence intensity from different groups (*n* = 6). (I to K) Protein expression levels of GPX4 and xCT in mouse lungs across treatment groups. Data were evaluated by one-way ANOVA with Holm–Sidak post hoc tests. *P < 0.05, **P < 0.01, ***P < 0.001, and ****P < 0.0001. Scale bars, 50 μm.

To elucidate the protective mechanisms of QFGB in COPD, we investigated its effects on oxidative stress and ferroptosis pathways. Oxidative stress is a critical pathogenic factor in COPD progression, characterized by persistent accumulation of ROS, including superoxide anion (O_2_^•−^), hydroxyl radical (·OH), hydrogen peroxide (H₂O₂), and lipid peroxides, leading to disrupted redox homeostasis and systemic oxidative damage [21]. Notably, GPX4 plays a pivotal role in GSH-dependent lipid peroxide reduction, and xCT regulates GSH biosynthesis through cystine/glutamate exchange. Dysregulation of this Xc^−^/GSH/GPX4 axis leads to impaired lipid peroxide clearance and subsequent ferroptosis induction (Fig. 4A) [8]. Therefore, we first examined the levels of these antioxidants and the malondialdehyde (MDA), and found that QFGB reversed the levels of GSH, SOD, CAT, and MDA in COPD mice (Fig. 4B to D and Fig. S4). We also observed that QFGB treatment reduced the accumulation of ROS and Fe^2+^ in the lungs (Fig. 4E to H). Next, we detected the protein expression of GPX4 and xCT. The results showed that QFGB increased the expression of GPX4 and xCT in the lungs of COPD mice, preventing ferroptosis (Fig. 4I to K). These findings further demonstrate that QFGB exerts a therapeutic effect on COPD mice by mitigating pulmonary oxidative stress and ferroptosis, thereby alleviating lung tissue damage.

### Immunomodulatory effects of QFGB in COPD mice

**Fig. 5.**
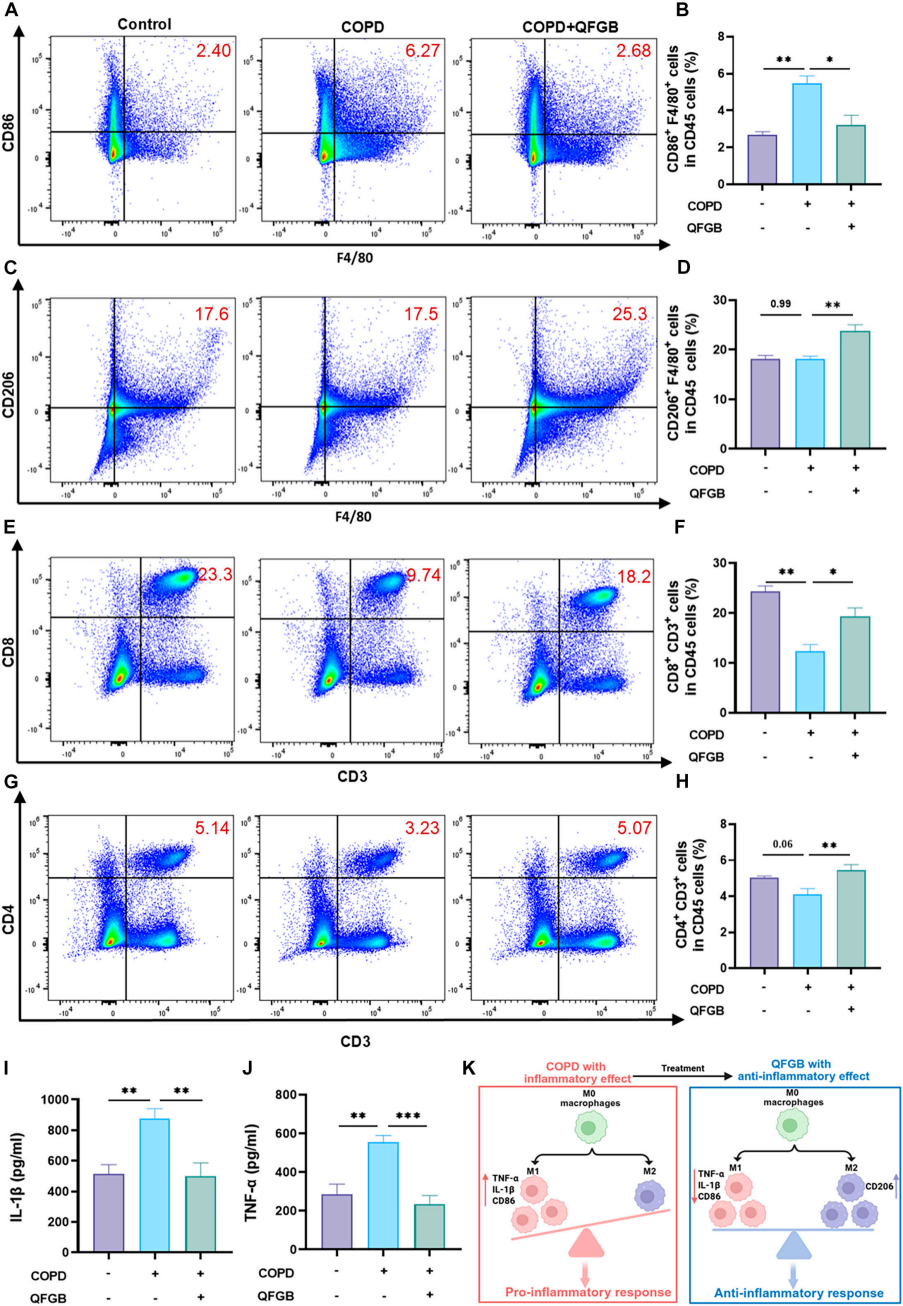
QFGB modulates immune responses in a murine COPD model. (A to D) Flow cytometry analysis of M1 and M2 macrophages in lung tissues across experimental groups (*n* = 3). (E to H) Flow cytometry analysis of T cells in lung tissues across experimental groups (*n* = 3). (I and J) Serum levels of inflammatory cytokines IL-1β and TNF-α measured by ELISA in different groups (*n* = 5). (K) Schematic diagram of M1 and M2 macrophage polarization in COPD mice. Data were evaluated by one-way ANOVA with Holm–Sidak post hoc tests. *P < 0.05, **P < 0.01, and ***P < 0.001.

Chronic airway inflammation, a hallmark feature of COPD, can propagate into systemic circulation, leading to widespread inflammatory responses. As the largest secondary lymphoid organ in mammals, the spleen serves as a critical indicator of overall immune status [22]. To investigate the immunoregulatory mechanisms of QFGB in COPD, we performed comprehensive flow cytometric analysis of splenic macrophage polarization. The results showed that COPD mice exhibited increased M1 macrophage polarization, decreased M2 polarization, and an elevated M1/M2 ratio, which was restored after QFGB treatment (Fig. 5A to D and Figs. S5 to S7). We also quantified T cell populations in our analysis. CD4 T cells, which perform multifaceted functions by differentiating into various helper T cell subsets to facilitate broad-spectrum immune responses [23], along with CD8 T cells—the “commanders” of adaptive immunity that execute crucial cytotoxic functions to eliminate invaders—were both significantly reduced in COPD mice [24], indicating compromised immune function. Notably, QFGB treatment effectively restored these T cell populations (Fig. 5E to H and Figs. S8 and S9). Furthermore, QFGB reduced serum levels of pro-inflammatory cytokines in COPD mice (Fig. 5I and J). The increased M1/M2 macrophage ratio promoted pro-inflammatory cytokine, exacerbating systemic inflammation—a trend reversed by QFGB (Fig. 5K). These findings suggest that QFGB alleviates immune imbalance caused by COPD by rebalancing M1 and M2 macrophage polarization, modulating T cell-mediated immunity, reducing inflammation, and enhancing immune function.

### QFGB regulate M1 and M2 macrophage polarization in COPD mouse lungs

**Fig. 6.**
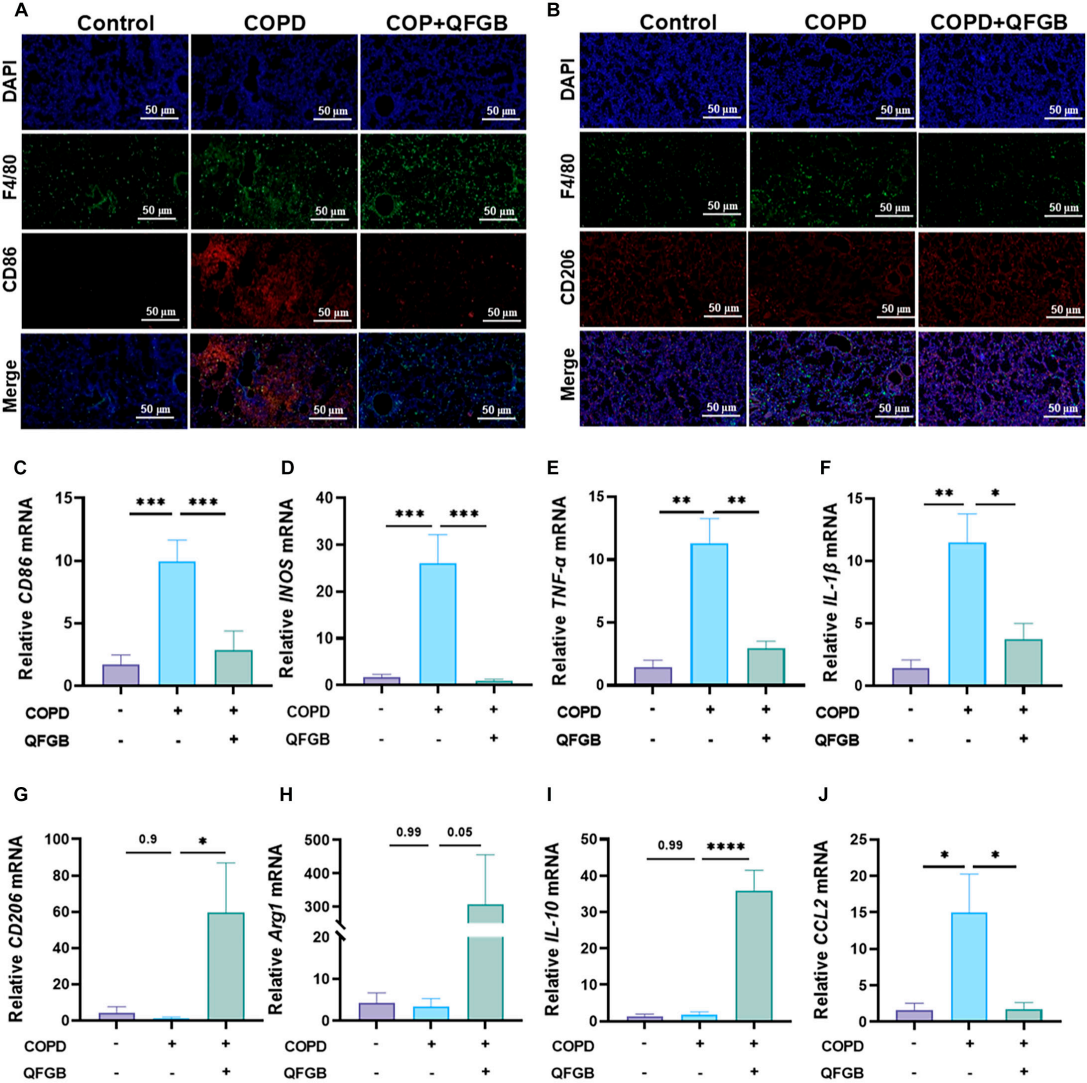
QFGB alleviates pulmonary and intestinal inflammation in COPD mice. (A and B) Immunofluorescence staining of M1 and M2 macrophages in lung tissues across experimental groups (*n* = 6). (C to J) Relative mRNA levels of M1 and M2 macrophage markers, inflammatory factors, and chemokines in lung tissues (*n* = 3 to 6). Data were evaluated by one-way ANOVA with Holm–Sidak post hoc tests. *P < 0.05, **P < 0.01, ***P < 0.001, and ****P < 0.0001. Scale bars, 50 μm.

Following the discovery that QFGB modulates systemic M1 and M2 macrophage polarization, we further validated these effects by examining macrophage polarization in lung tissues. Using immunofluorescence and qRT-PCR, we assessed the relative fluorescence intensity of M1 and M2 macrophage markers and their associated mRNA expression levels. Consistent with previous findings, QFGB administration reduced the proportion of CD86 in F4/80 macrophages (M1) in the lungs of COPD mice (Fig. 6A and Fig. S10) and increased the proportion of CD206 in F4/80 macrophages (M2) (Fig. 6B and Fig. S11). The mRNA expression of M1 macrophage markers *CD86* and *INOS* and related cytokines *TNF-α* and *IL-1β* was decreased (Fig. 6C to F), while the mRNA expression of M2 macrophage markers *CD206* and *Arg1* and related cytokine *IL-10* was increased (Fig. 6G to I). Additionally, we observed reduced mRNA expression of pro-inflammatory chemokines *CCL2*, *CCL6*, and *MIP-1β* (Fig. 6J and Fig. S12A and B). These results align with our in vitro findings, demonstrating that QFGB can inhibit M1 polarization of macrophages. However, unlike the in vitro results, our in vivo experiments revealed that QFGB promotes M2 polarization of macrophages, thereby reducing the M1/M2 ratio. These findings further confirm that QFGB ameliorates the polarization of M1 and M2 macrophage in the lungs of COPD mice.

### QFGB regulate gut microbiota and intestinal M1 and M2 macrophage polarization in COPD mice

**Fig. 7.**
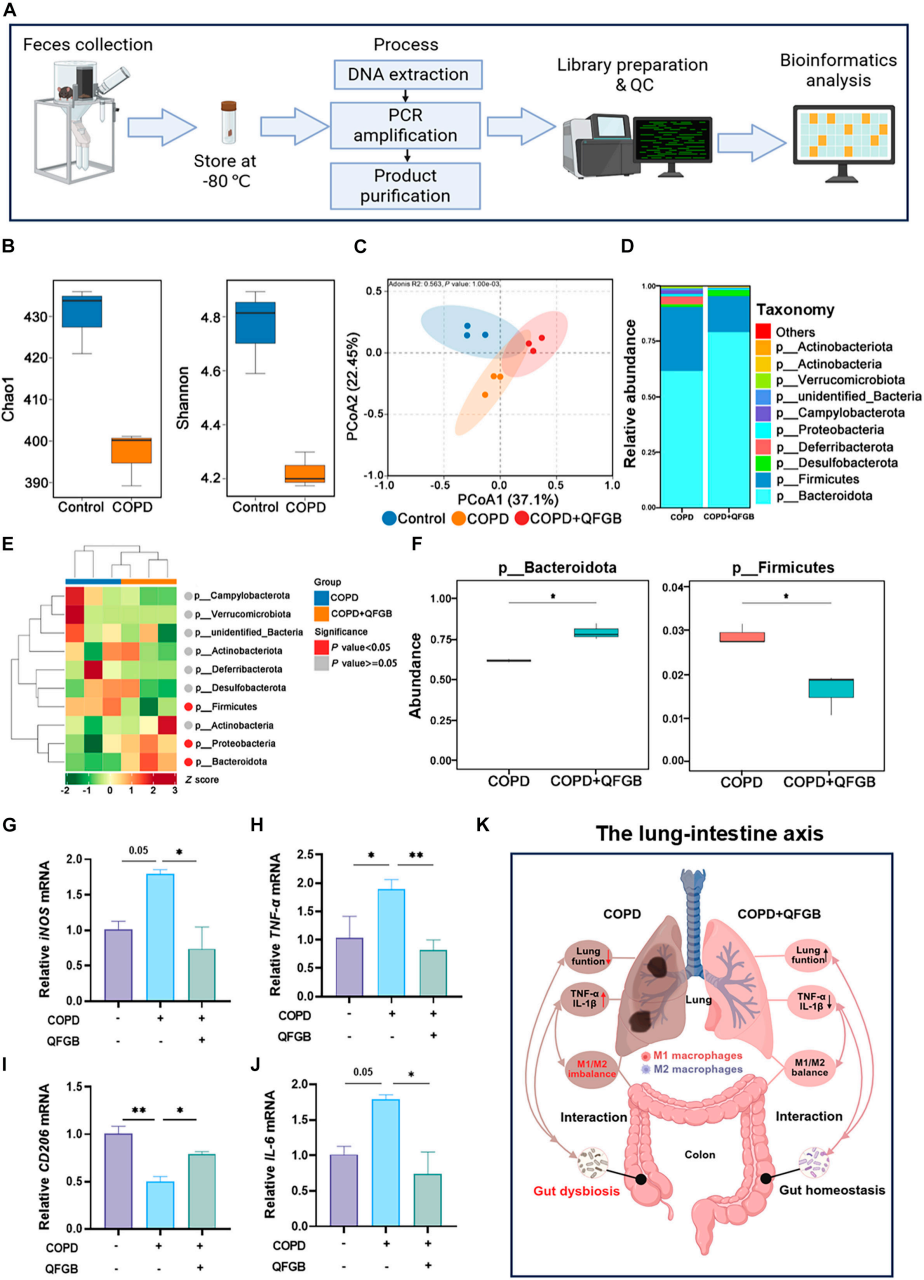
QFGB modulates gut microbiota and the polarization of macrophage. (A) Flowchart of 16*S* rRNA sequencing sample analysis procedure. (B) Intergroup difference analysis of alpha diversity indices between normal mice and COPD mice based on 16*S* rRNA sequencing. (C) PCoA plot demonstrating similarity in microbial community structure among samples. (D) Bar chart showing relative abundance of phylum at genus level. (E) Phylum-level heatmap of differentially abundant taxa between groups analyzed by Metastats, showing species with significant differences. (F) Phylum-level boxplot of differentially abundant taxa between groups analyzed by Metastats. (G to J) Relative mRNA levels of M1 and M2 macrophage markers and inflammatory factors in intestinal tissues (*n* = 3 to 6). (K) Schematic diagram of lung–gut axis interaction. Data were evaluated by one-way ANOVA with Holm–Sidak post hoc tests. *P < 0.05, **P < 0.01, and ***P < 0.001.

The “gut–lung axis” refers to the bidirectional communication pathway between gut microbiota and lung tissue, where alterations in gut microbiota are closely associated with pulmonary diseases such as COPD [25]. To investigate changes in gut microbiota of COPD mice following QFGB administration, we performed 16*S* rRNA sequencing analysis of fecal samples (Fig. 7A). Alpha diversity analysis revealed lower Chao1 and Shannon indices in COPD mice (Fig. 7B and Fig. S13A), indicating reduced species richness and diversity in the gut microbiota. Beta diversity analysis demonstrated distinct separation between groups in PCoA plots (Fig. 7C), suggesting significant microbial structural changes.

A decreased p__Firmicutes/p__Bacteroidetes ratio in the gut microbiota indicates reduced systemic inflammation [26]. We analyzed phylum-level relative abundance in COPD and QFGB groups based on taxonomic annotation (Fig. 7D). Metastats analysis of intergroup species abundance at the phylum level (Fig. 7E and F) showed that QFGB treatment reduced p__Firmicutes abundance while increasing p__Bacteroidetes in COPD mice. At the species level, significant differences were observed in s__Burkholderiales_bacterium_YL45 and s__Lactobacillus_johnsonii (Fig. S13B and C), suggesting potential associations with QFGB intervention, although conclusive evidence remains to be established. These results demonstrate significant alterations in gut microbiota of COPD mice and the regulatory effects of QFGB.

Based on 16*S* rRNA sequencing findings, we hypothesized that M1 and M2 macrophage polarization might also occur in the intestinal tract, potentially triggering inflammatory cascades. qRT-PCR analysis of colon tissue revealed that, similar to pulmonary findings, QFGB administration decreased mRNA expression of *INOS*, *TNF-α*, and *IL-6* while increasing *CD206* and *Arg1* expression in COPD mice (Fig. 7G to J and Fig. S14). Collectively, these results suggest that QFGB may alleviate intestinal inflammation by modulating gut microbiota and restoring intestinal M1 and M2 macrophage polarization (Fig. 7K).

### QFGB ameliorate intestinal pathology and systemic inflammation in IBD mice

**Fig. 8.**
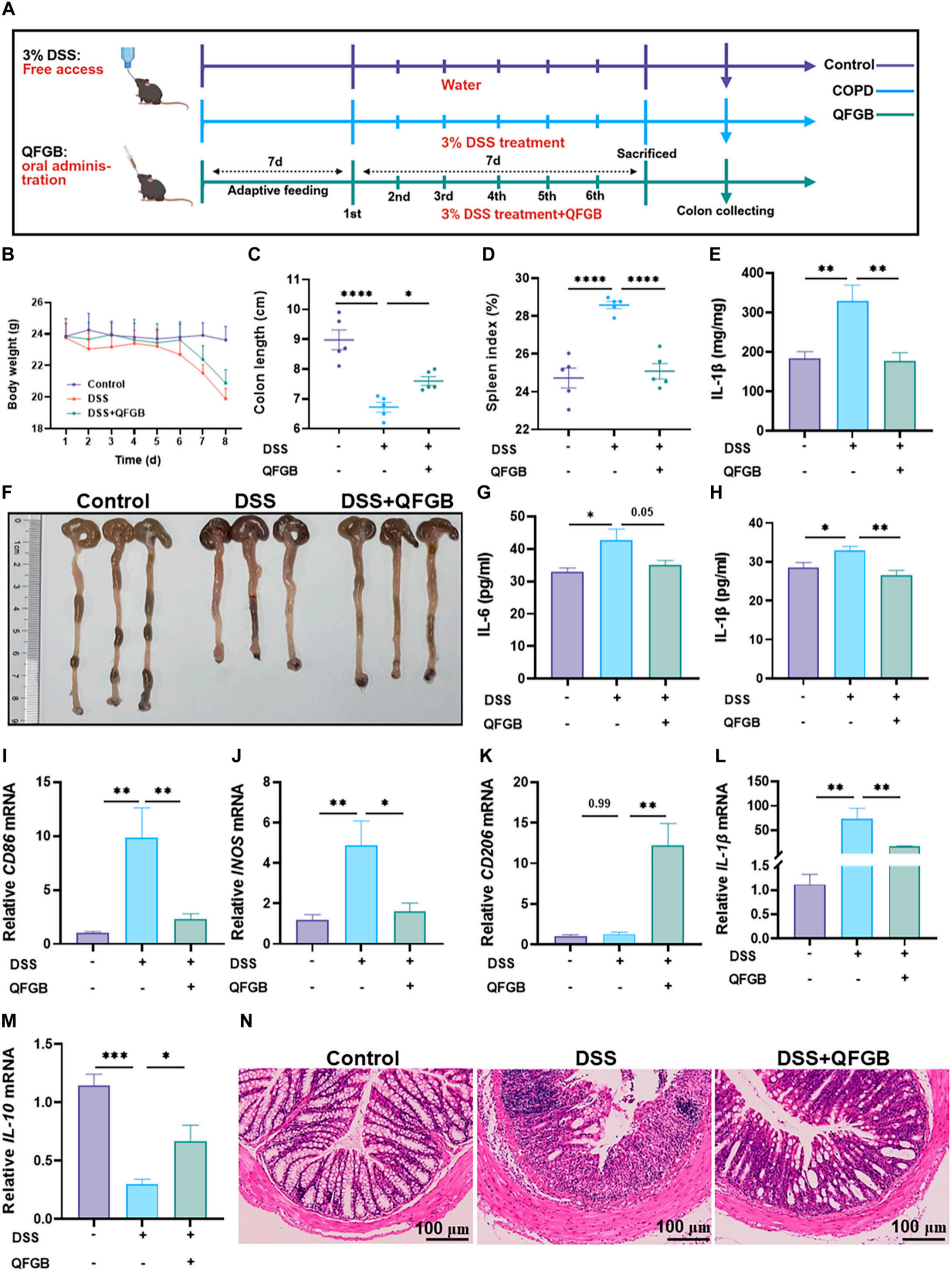
QFGB ameliorates intestinal lesions and systemic inflammation in mice with colitis. (A) Schematic diagram of DSS-induced mouse model establishment and drug administration protocol. (B) Line graph of body weight changes in different groups (*n* = 5). (C) Comparison of colon lengths among groups (*n* = 5). (D) Splenic index (spleen-to-body weight ratio) comparison across groups (*n* = 5). (E) Intestinal levels of inflammatory cytokine IL-1β measured by ELISA (*n* = 5). (F) Representative images of colons from each experimental group (*n* = 3). (G and H) Serum levels of inflammatory cytokines IL-6 and IL-1β measured by ELISA (*n* = 4 to 6). (I to M) Relative mRNA levels in intestinal tissues of M1 and M2 macrophage markers and inflammatory factors (*n* = 3 to 6). (N) H&E staining of intestinal sections. Data were evaluated by one-way ANOVA with Holm–Sidak post hoc tests. *P < 0.05, **P < 0.01, and ***P < 0.001. Scale bars, 100 μm.

To investigate the therapeutic efficacy of QFGB on intestinal inflammation, we established a DSS-induced IBD mouse model (Fig. 8A). Compared with the control group, DSS-treated mice exhibited significant weight loss, reduced colon length, and elevated spleen index. These pathological changes were significantly ameliorated by QFGB treatment (Fig. 8B to D and F). Inflammatory factor analysis revealed that QFGB suppressed the elevation of pro-inflammatory cytokines IL-1β and IL-6 in the serum of IBD mice (Fig. 8G and H). Consistent results were observed in enzyme-linked immunosorbent assay (ELISA) assays measuring IL-1β levels in colon tissues (Fig. 8E). Subsequent qRT-PCR analysis of colon tissues demonstrated that QFGB treatment decreased mRNA expression of M1 macrophage markers and related cytokines while increasing expression of M2 markers and related cytokines (Fig. 8I to M and Fig. S15A and B), suggesting that QFGB could modulate the polarization of M1 and M2 macrophages in the intestine. Histopathological examination further confirmed that QFGB alleviated colonic damage, including structural disruption, crypt destruction, focal erosion, ulceration, and inflammatory cell infiltration (Fig. 8N). Collectively, these findings demonstrate that QFGB could also modulate the systemic inflammatory microenvironment by alleviating intestinal inflammation.

## Discussion

Our previous studies have demonstrated that QFGB primarily contains flavonoids, organic acids, polysaccharides, and other components with immunomodulatory properties [27]. The major bioactive constituents entering systemic circulation predominantly exhibit anti-inflammatory, antioxidant, and immunoregulatory effects [28]. Current studies have demonstrated the therapeutic efficacy of QFGB in treating respiratory diseases including pneumonia and allergic rhinitis. Wang and colleagues [29] revealed that QFGB potentially exerts pulmonary protective effects by suppressing *Streptococcus pneumoniae*-induced inflammatory responses through inhibition of the Toll-like receptor 4 (TLR4)/nuclear factor κB (NF-κB) signaling pathway. Similarly, Zhang [30] reported that QFGB alleviates pathological damage in allergic rhinitis rat models by modulating the T helper 1 (Th1)/Th2/Th17 immune balance. However, its therapeutic effects and underlying mechanisms in COPD remain unclear. In this study, we found that QFGB-containing serum exhibited antioxidant and macrophage polarization-modulating effects in vitro, which inspired subsequent in vivo experiments in COPD mice.

COPD, a prevalent and complex respiratory syndrome, is characterized by airway obstruction and systemic manifestations, with chronic bronchitis and emphysema as its primary clinical phenotypes [31]. Its pathogenesis involves epithelial injury, oxidative stress, inflammation, innate/adaptive immune responses, and tissue remodeling. We investigated the therapeutic mechanisms of QFGB for COPD through 2 pathways: first, by mitigating pulmonary injury caused by oxidative stress-induced ferroptosis; second, by regulating COPD-associated immune microenvironment disruption via the gut–lung axis.

Oxidative stress represents one of the 4 fundamental mechanisms in COPD pathogenesis. Beyond its direct damaging effects, it can also potentiate the other 3 mechanisms [32]. Exogenous ROS accumulate in the body and disrupt the endogenous oxidant/antioxidant balance. They also alter protein production and enzyme activity through epigenetic regulation in cells (e.g., bronchial alveolar epithelial cells and macrophages) [9]. Under sustained oxidative stress, decreased xCT expression and severe GSH depletion reduce GPX4 activity, impairing the conversion of LOOH to LOH. This leads to lipid peroxide accumulation and triggers ferroptosis [33]. Under these conditions, lung cells are damaged, and pulmonary vascular endothelial cell apoptosis is induced, ultimately resulting in necrosis and emphysema [34]. In this study, we established a mouse model of emphysema by administering LPS and ELT once weekly for 4 weeks. This model replicates key pathological features of COPD, including impaired lung function, emphysema, tissue damage, and airway inflammation. Notably, emphysema and inflammation persisted for at least 2 months after treatment cessation [35]. We employed this model to evaluate the therapeutic efficacy of QFGB in COPD, assessing the degree of emphysema, oxidative stress injury, and ferroptosis. Our findings demonstrate that QFGB reduces oxidative stress and ferroptosis in lung tissue of COPD.

The chronic inflammation in COPD is driven by the activation of both innate and adaptive immune pathways [36]. Macrophage infiltration is a hallmark of COPD lungs, playing pivotal roles in chronic inflammation, with abundance correlating with emphysema severity [12]. The polarization of pro-inflammatory M1 macrophages and anti-inflammatory M2 macrophages plays a pivotal role in modulating chronic inflammation in COPD pathogenesis. [37]. T cell responses represent a critical component of the immune system. Upon antigen recognition, T cells become activated and exert their immune functions through proliferation and differentiation [38]. In this study, we demonstrate that QFGB can modulate the immune microenvironment and alleviate chronic inflammation in COPD, based on observed effects on macrophage polarization and T cell proliferation/differentiation in a murine COPD model.

The lung–gut axis has garnered growing attention, as the stability of intestinal microecology—namely, the homeostasis of intestinal microorganisms—plays a pivotal role in maintaining lung health. Intestinal microbial dysbiosis can trigger a range of pulmonary diseases via the gut–lung axis, including viral pneumonia, asthma, tuberculosis, and COPD [15]. Clinical evidence has corroborated the existence of a pathophysiological link between the lungs and the large intestine. In summary, COPD is a complex disease often accompanied by comorbidities such as intestinal disorders. With the discovery of how intestinal health influences pulmonary immunity, the concept of the “lung–gut axis” has come into focus [39]. This bidirectional crosstalk between the gut and lungs in COPD reveals that COPD can induce intestinal inflammation, barrier impairment, hypoxia, oxidative stress, as well as microbiota dysbiosis and alterations in the production of microbial metabolites.

In COPD patients, injured pulmonary/intestinal cells communicate via elevated pro-inflammatory mediators, exacerbating disease by stimulating innate immunity [40]. Emerging evidence suggests that airborne pollutants may dysregulate immune function, exacerbate inflammation and oxidative stress, and alter gut–lung axis signaling, although research remains limited [41]. Gut microbiota critically modulates pulmonary immunity through the gut–lung axis [14]. Research has demonstrated that the gut microbiota–lung COPD axis was connected. A potentially beneficial bacterial strain and its functional component may be developed and used as alternative agents for COPD prevention or treatment [42]. Microbial composition shifts can impair infection resistance and increase inflammatory disease susceptibility by altering immune cell populations [43]. It has been reported that in rat models of chronic obstructive pulmonary disease (COPD), there is an imbalance in Th17/regulatory T cells (Tregs), accompanied by dysbiosis of the intestinal and pulmonary microbiota in terms of abundance, diversity, and community structure [44]. Given the gut–lung axis’s significance in COPD, elucidating drug mechanisms targeting this axis is crucial for COPD treatment. In this study, after identifying the immunomodulatory effects of QFGB, we shifted our focus from the lungs to the large intestine. We found that the gut microbiota homeostasis was altered in COPD mice, and QFGB could modulate the gut microbiota in these mice. These results led us to further explore the gut–lung axis theory. We discovered that QFGB also regulates macrophage polarization in the colon, and therapeutic experiments in mice with enteritis provided additional compelling evidence that QFGB can alleviate intestinal damage and modulate intestinal macrophage polarization. Based on these findings, we demonstrated that QFGB mitigates COPD-induced intestinal inflammation and injury, and alleviates chronic inflammation caused by COPD by regulating the immune microenvironment through the gut–lung axis.

However, our study still has some limitations. How does QFGB regulate the gut microbiota? Is there a connection between the gut microbiota and macrophage polarization? These questions require further in-depth exploration in subsequent research.

**Fig. 9.**
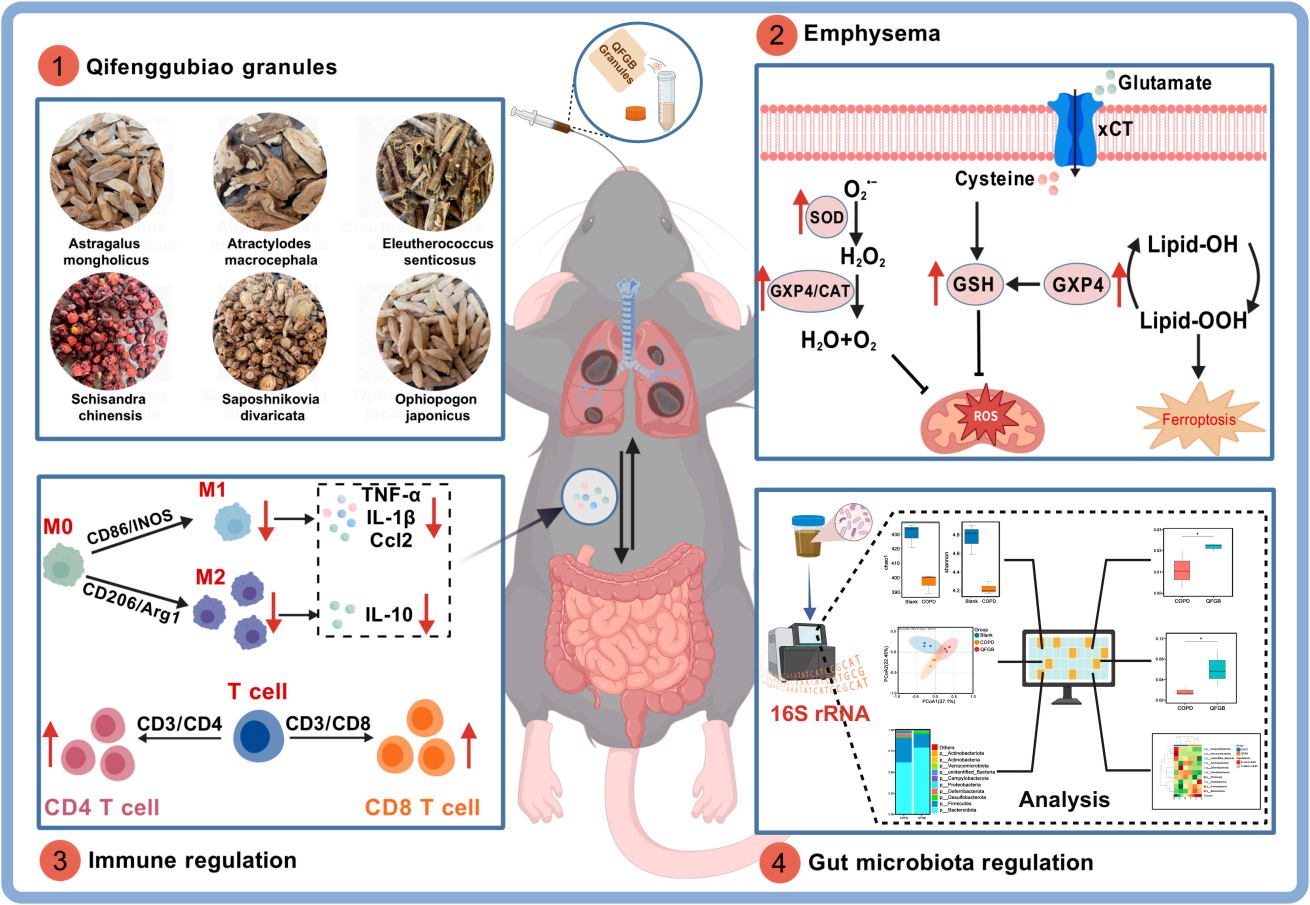
QFGB alleviates pulmonary oxidative stress and ferroptosis in COPD, while modulating the immune microenvironment via the gut–lung axis to suppress chronic inflammation.

In summary, this study demonstrates the therapeutic potential of QFGB for COPD, showing its ability to alleviate pulmonary oxidative stress and ferroptosis in COPD while also modulating the immune microenvironment through the gut–lung axis to suppress chronic inflammation (Fig. 9). As a novel traditional Chinese medicine, our findings fully highlight the therapeutic potential of QFGB for COPD and provide new evidence supporting drug intervention via the gut–lung axis in COPD treatment.

## Data Availability

The authors declare that all data supporting the findings of this study are available within the paper and its Supplementary Materials files.
