# Supplementary material for: Investigating the Mechanism of Qifenggubiao Granules in COPD Treatment: An Integrated Exploration of Ferroptosis and the Gut–Lung Axis
Source: Biomater Res. 2025 Oct 8;29:0263. doi: 10.34133/bmr.0263 (PMC12504822; doi:10.34133/bmr.0263)
Supplement: Supplementary 1 — Materials and Methods Figs. S1 to S15 Tables S1 and S2 [file bmr.0263.f1.docx]

**Supporting information**

**Investigating the Mechanism of Qifenggubiao Granules in COPD Treatment: An Integrated Exploration of Ferroptosis and the Gut-Lung Axis**

Mianfeng Zheng^1, #^, Lixin Huang^1, #^, Haitao Yuan^2, 4, *^, Zhuoya Li^3^, Yi Wang^1^, Yangjing Su^4^, Zhixin Deng^4^, Ali Chen^4^, Weiguo Zhao^3, *^, Weiming Wang^5, *^, Wei Xiao^1, *^

^1^Key Laboratory of Glucolipid Metabolic Disorder, Ministry of Education, Guangdong Pharmaceutical University, Guangzhou, 510006, China

^2^Department of nuclear medicine, First School of Clinical Medicine, The First Affiliated Hospital of Guangdong Pharmaceutical University, 19 Nonglinxia Road, Yuexiu District, Guangzhou, 510080, China

^3^Department of Pharmacy, Zhongshan People’s Hospital, Zhongshan, 528404, China

^4^Center for Drug Research and Development, Guangdong Provincial Key Laboratory for Research and Evaluation of Pharmaceutical Preparations, Guangdong Pharmaceutical University, Guangzhou, 510006, China

^5^Institute of Chinese Materia Medica, Heilongjiang Academy of Chinese Medicine Sciences, No.72, Xiang’an Street, Xiangfang District, Harbin 150036, Heilongjiang, China

^#^These authors contributed equally

***Corresponding authors:** Wei Xiao (xw7688@smu.edu.cn), Weiming Wang (zyyjy@163.com), Weiguo Zhao (1274413730@qq.com) and Haitao Yuan (yht193525@163.com).

**Materials and Methods**

**Cell Viability Assay**

Beas-2B cells and RAW 264.7 macrophages were cultured in DMEM medium supplemented with 10% FBS and 1% penicillin/streptomycin. The cells were seeded in a 96-well plate at a density of 8×10³ cells per well, with 100 μL of medium added to each well. The plate was then incubated for 24 hours. After 80% of the cells adhered to the well walls, culture medium containing H₂O₂ (0, 250, 500, 750, and 1000 μM) or LPS (0, 1, 2, 5, and 10 μM) at gradient concentrations was added and incubated for another 24 hours. Subsequently, 10 μL of CCK-8 reagent (Meilunbio) was added to each well, followed by incubation for 1 hour. Finally, the optical density (OD) at 450 nm was measured using a microplate reader (Agilent Technologies, Inc.) to calculate the inhibition rate and half-maximal inhibitory concentration (IC₅₀) for Beas-2B cells and RAW 264.7 macrophages.

**Enzyme-linked immunosorbent assay**

Colon tissues were homogenized, and proteins were extracted using RIPA lysis buffer. The levels of inflammatory cytokines were measured using ELISA kits from NeoBioscience (China), including IL-6 (Cat. No.: EMC004.96), TNF-α (Cat. No.: EMC102a.96), and IL-1β (Cat. No.: EMC001b.96), following the manufacturer’s protocols strictly to quantitatively assess the degree of inflammation.

**Flow Cytometry**

Kit (Solarbio, P8630, Beijing, China) used towasseparate lymphocytes according to the manufacturer’ sprotocol. mice spleens were removed under aseptic conditions, and the red blood cells in the suspension were removed by treatment with PBS (PH7.2) solution prior to preparation of single-cell suspension of splenocytes. M1 macrophage were labeled with CD45-PE, F4/80- APC, CD86-APC-Cy7, M2 macrophage were labeled with CD45-PE-CF594, F4/80- APC, CD206-APC-Cy7, CD4 helper T cells were labeled with CD45-PE-CF594, CD3-FITC, CD4-APC, CD8 cytotoxic T cells were labeled with CD45-PE-CF594, CD3-FITC, CD8-APC-Cy7. Then, 2 uL cocktail stimulation solution with GolgiStop protein inhibitor were added to the lymphocytes, and the mixture was gently mixed and incubated in the darkat 37 ℃ for 5 h. The cell surface was stained with monoclonal antibody, and the cells were fixed and permeabilized. Intracellularstaining was performed. Macrophage and T Cells were detectedvia flow cytometry.

**Quantification and Statistical Analysis**

All data are presented as mean ± SEM and were compared using two-tailed unpaired Student’s t-test or one-way ANOVA with Holm–Sidak post hoc tests. Significance levels were set at **p <* 0.05, ***p <* 0.01, and ****p <* 0.001.

**
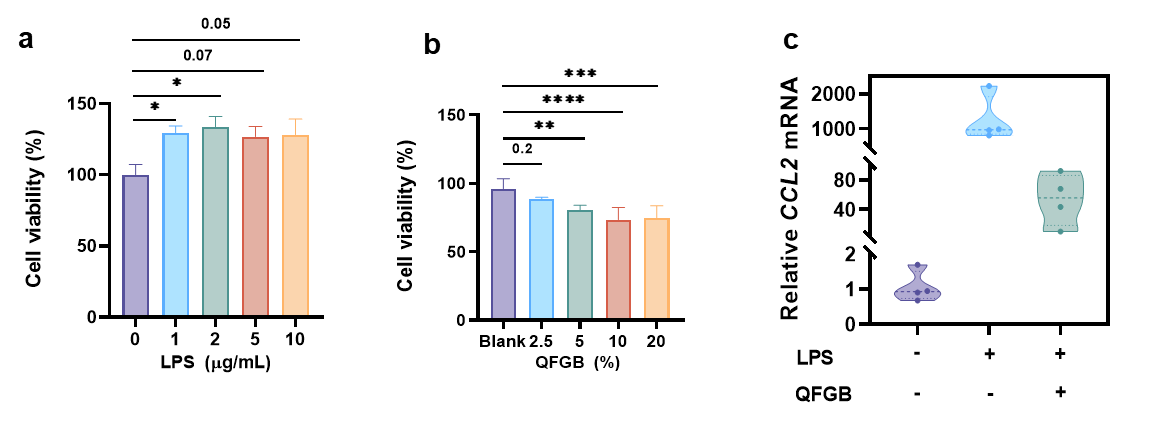
**

**Figure S1** (a, b) CCK-8 assay examining the effects of LPS and QFGB-containing serum on RAW264.7 cells (n=6). (c) Relative mRNA levels of M1 macrophage-associated chemokines in RAW264.7 cells (n=4). Data were represented as mean ± SEM and were evaluated using one-way ANOVA with Holm–Sidak post hoc tests. **p* < 0.05 and ***p* < 0.01.


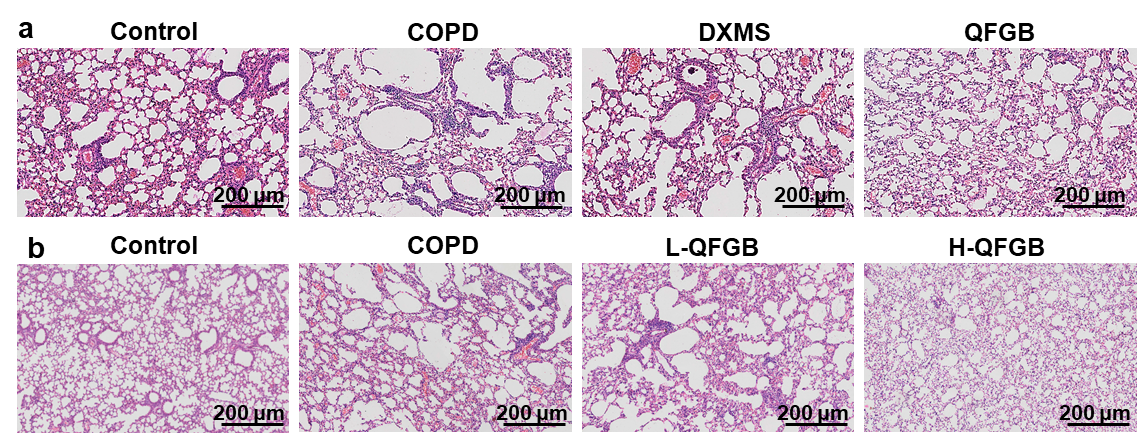


**Figure S2** (a) H&E staining of lung tissues (including dexamethasone treatment group), (b) H&E staining of lung tissue (including the low-dose QFGB treatment group), Scale bars, 200 μm.

**Figure S3** Quantification of Tunel-positive apoptotic cells in lung sections (n=6). Data were represented as mean ± SEM and were evaluated using one-way ANOVA with Holm–Sidak post hoc tests. ****p* < 0.001.


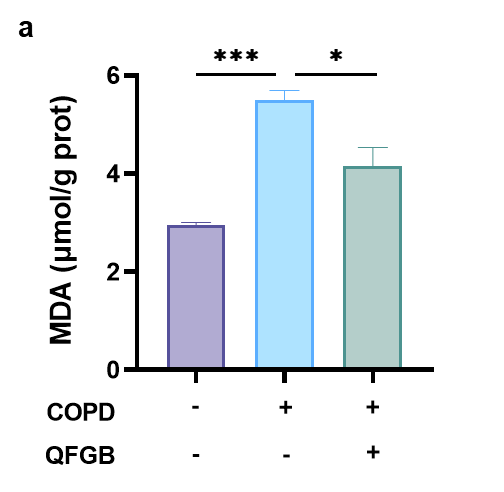


**Figure S4** Levels of MDA in mouse lungs (n=3). Data were represented as mean ± SEM and were evaluated using one-way ANOVA with Holm–Sidak post hoc tests. **p* < 0.05, and ****p* < 0.001.


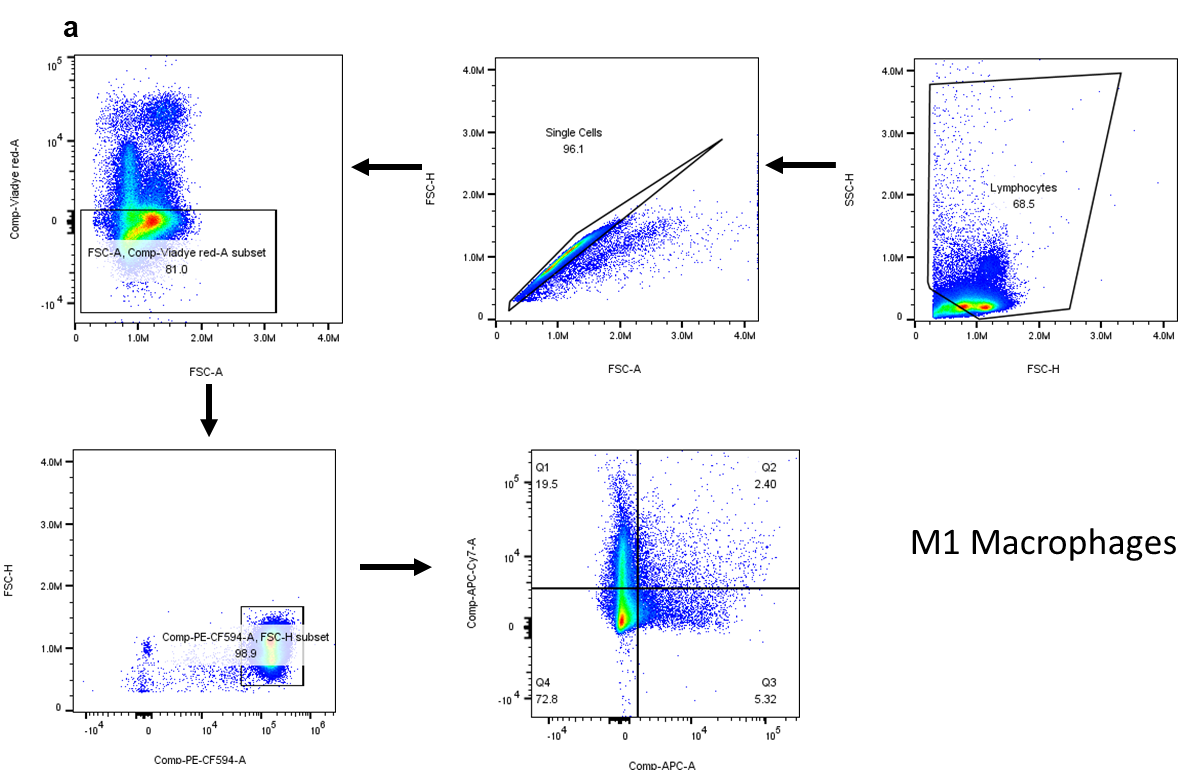


**Figure S5** Analytical procedure for M1 macrophage identification by flow cytometry.


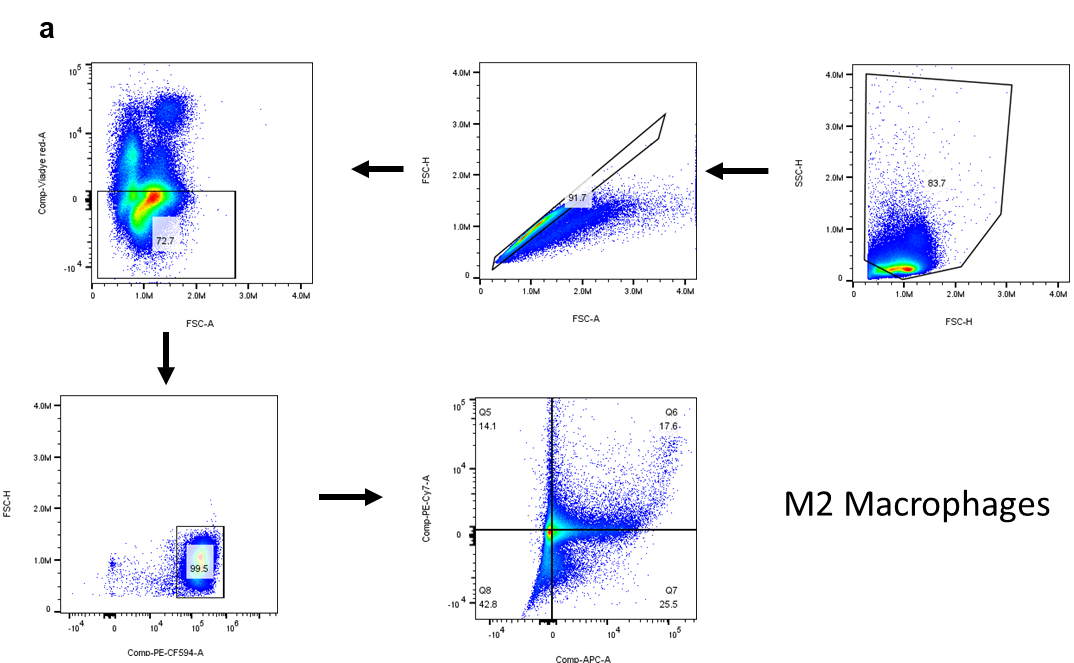


**Figure S6** Analytical procedure for M2 macrophage identification by flow cytometry.

**Figure.S7**. The ratio of M1and M2 macrophage (n=3). Data were represented as mean ± SEM and were evaluated using one-way ANOVA with Holm–Sidak post hoc tests. ****p* < 0.001.


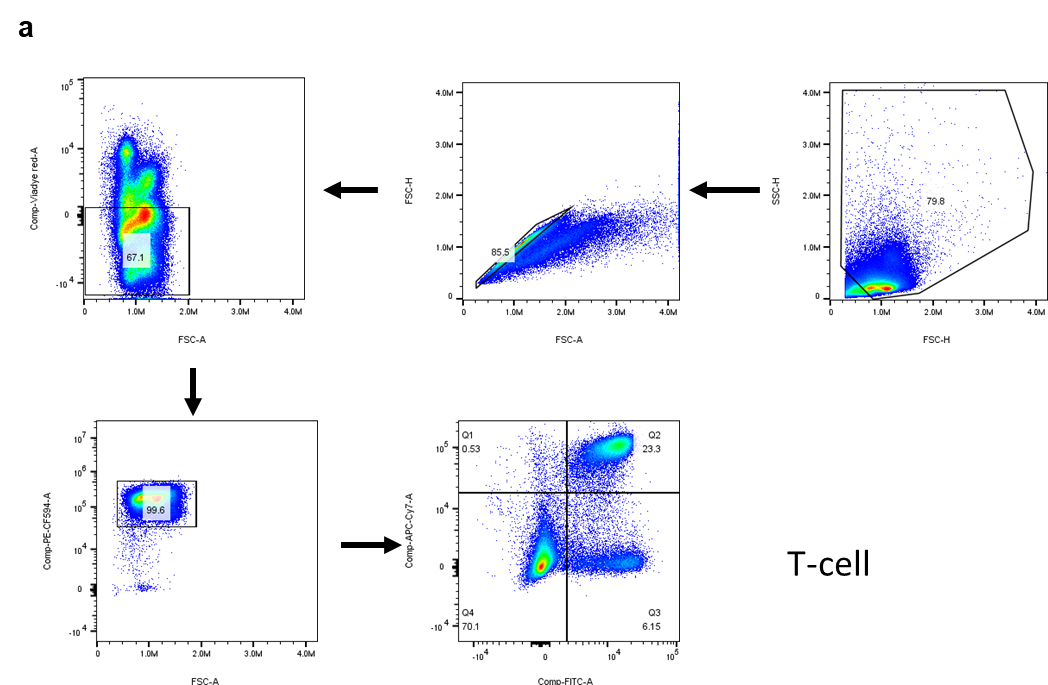


**Figure S8** Analytical procedure for CD8 T cell identification by flow cytometry.


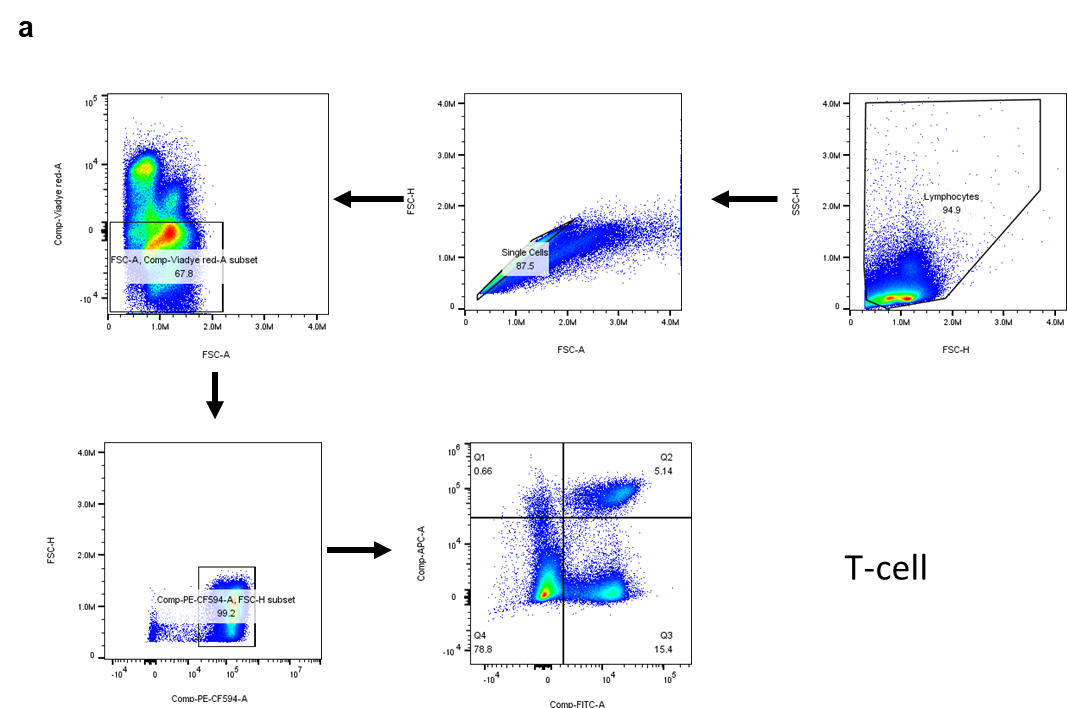


**Figure S9** Analytical procedure for CD4 T cell identification by flow cytometry.


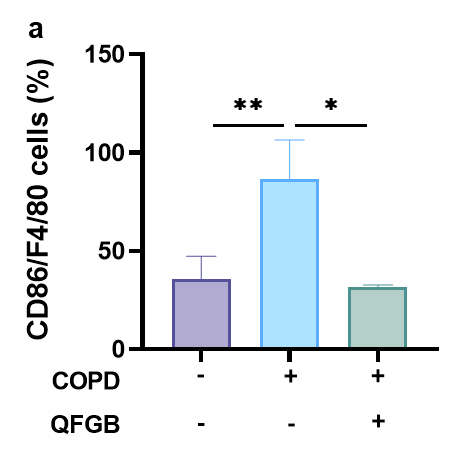


**Figure S10** Quantification of CD86/F4/80 cells as M1 polarization markers (n=6). Data were represented as mean ± SEM and were evaluated using one-way ANOVA with Holm–Sidak post hoc tests. **p* < 0.05 and ***p* < 0.01.


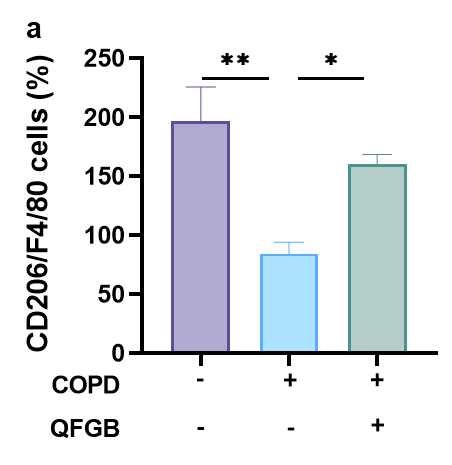


**Figure S11** Quantification of CD206/F4/80 cells as M2 polarization markers (n=6). Data were represented as mean ± SEM and were evaluated using one-way ANOVA with Holm–Sidak post hoc tests. **p* < 0.05 and ***p* < 0.01.


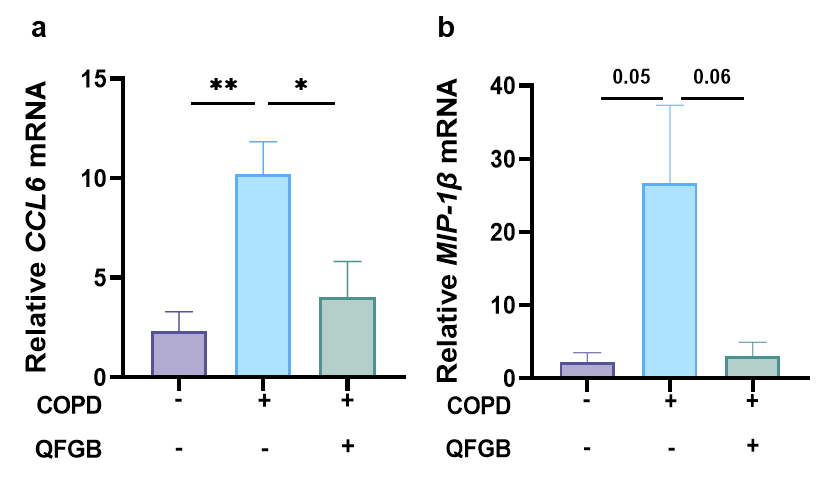


**Figure S12** (a, b) Relative mRNA levels of M1/M2 macrophage chemokines in lung (n=3-6). Data were represented as mean ± SEM and were evaluated using one-way ANOVA with Holm–Sidak post hoc tests. **p* < 0.05 and ***p* < 0.01.


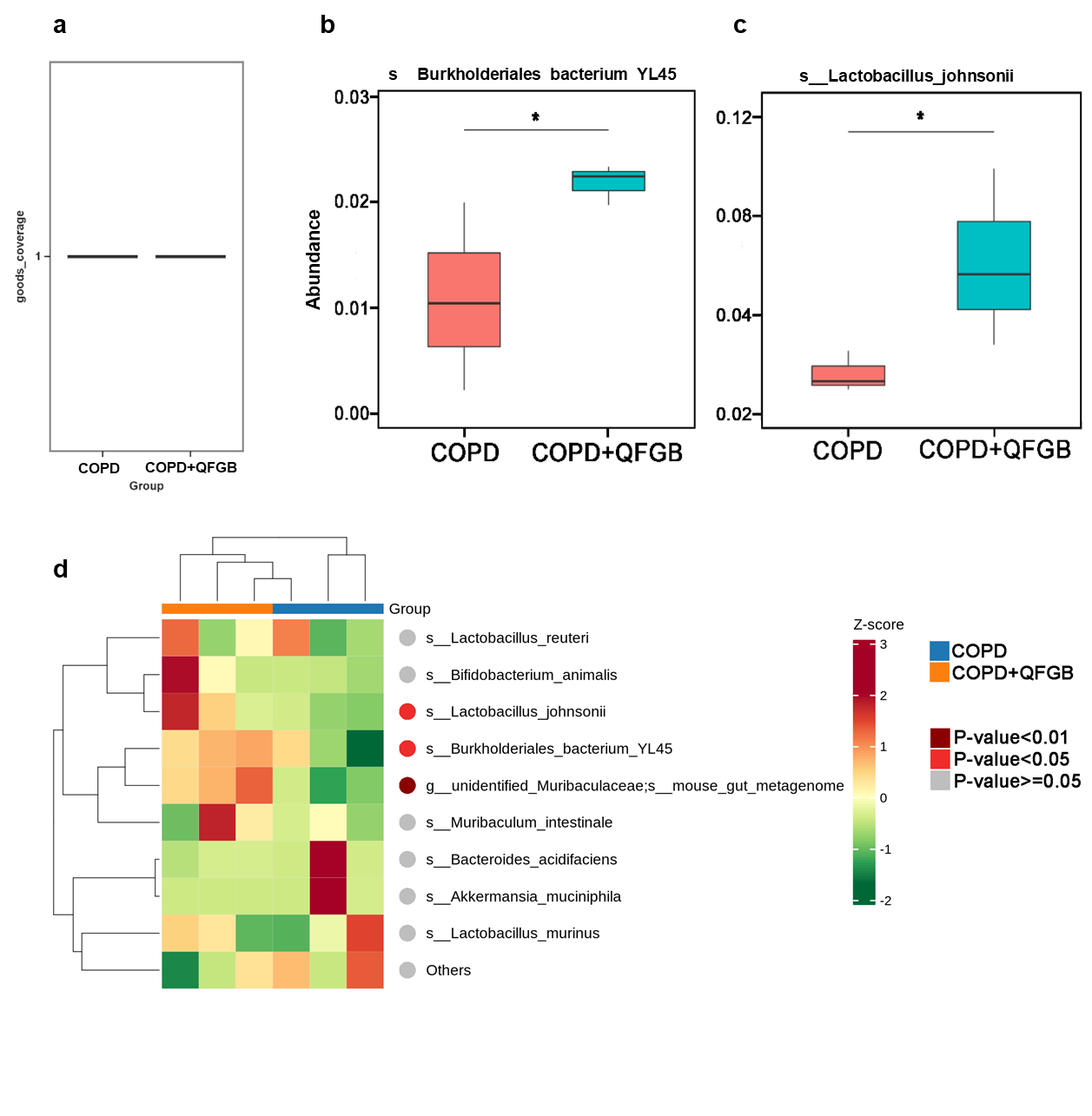


**Figure S13** (a) Goods_coverage index of alpha diversity intergroup difference analysis. (b, c) Species-level boxplot of differentially abundant taxa between groups analyzed by Metastats. (d) Phylum-level heatmap of differentially abundant taxa between groups analyzed by Metastats, showing species with significant differences.

**Figure S14** Relative mRNA levels of M1/M2 macrophage markers in intestinal tissues. (n=3). Data were represented as mean ± SEM and were evaluated using one-way ANOVA with Holm–Sidak post hoc tests. **p* < 0.05.


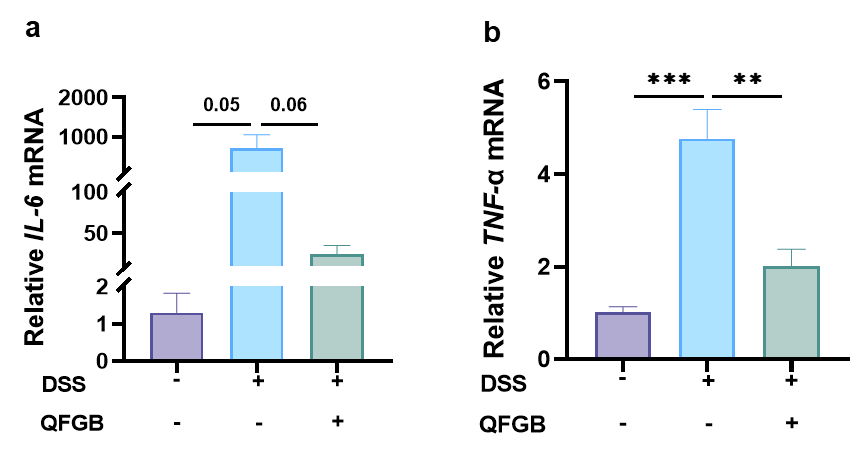


**Figure S15** (a, b) Relative mRNA levels of M1 and M2 macrophage inflammatory factors in intestinal tissues (n=4-5). Data were represented as mean ± SEM and were evaluated using one-way ANOVA with Holm–Sidak post hoc tests. **p* < 0.05, ***p* < 0.01, and ****p* < 0.001.

**Table S1**

**Primers for qRT‑PCR**

| ***Name*** | **Forward primer (5’-3’)** | **Reverse primer (5’-3’)** |
| --- | --- | --- |
| ***18S*** | GACACTGGAAGGATTGACA | TCTCGTTCGTTATCGGAATA |
| ***CD86*** | GTGACCTTGCTTAGACGTGC | CAATACGACTCGCAACCACA |
| ***CD206*** | CTTGTAGGAAGGAGGGTGGG | GGGTTCCATCACTCCACTCA |
| ***INOS*** | GTTGTGCATCGACCTAGGCT | CCCACCTCCAGTAGCATGTT |
| ***Arg1*** | GGACCTGGCCTTTGTTGATG | CCAGAGATGCTTCCAACTGC |
| ***IL-6*** | AGCCAGAGTCCTTCAGAGAGAT | AGCCAGAGTCCTTCAGAGAGAT |
| ***IL-10*** | GCTGGACAACATACTGCTAACCG | CACAGGGGAGAAATCGATGACAG |
| ***TNF-α*** | AGTTCCAACGCTACCTGAGTG | GGAGGAGGTAGGCACAACTT |
| ***IL-1β*** | TCAGGCAGGCAGTATCACTC | AGCTCATATGGGTCCGACAG |
| ***CCL2*** | CAGCAGGTGTCCCAAAGAAG | AAGTGCTTGAGGTGGTTGTG |
| ***MIP-1β*** | TTTCTCTTACACCTCCCGGC | ACTCATGTACTCAGTGACCCA |
| ***CCL6*** | GAAGATCGTCGCTATAACCC | ACATGGGATCTGTGTGGCAT |
| ***CXCL2*** | GCTGTTGTGGCCAGTGAACT | CTTCAGGGTCAAGGCAAACT |

**Table S2**

**Antibody information**

| **Antibodies** | **Sources** | **Identifier** | **Dilution ratios** |
| --- | --- | --- | --- |
| GPX4 antibody | Affinity | Cat# DF6701 | 1:1000 |
| xCT antibody | Abmart | Cat# T57046 | 1:1000 |
| F4/80 antibody | Servicebio | Cat#GB11027 | 1:5000 |
| CD86 antibody | Cell Signaling Technology | Cat#19589s | 1:100 |
| CD206 antibody | Servicebio | Cat#GB113497 | 1:500 |
| GAPDH polyclonal antibody | Affinity | Cat# 10494-1-AP | 1:5000 |
| Beta actin monoclonal antibody | Affinity | Cat# 66009-1-Ig | 1:5000 |
| Anti-rabbit IgG, HRP-linked antibody | Cell Signaling Technology | Cat# 7074 | 1:10000 |
| Alexa Fluor 594-conjugated goat anti-rabbit IgG | Jackson | Cat# 111-585-003 | 1:400 |
| Alexa Fluor 488-conjugated goat anti-rabbit IgG | Servicebio | Cat# GB25303 | 1:400 |
